# Supplementary material for: Are carbon emissions trading and green financial instruments synergistic? -Comprehensive quantitative research based on content analysis
Source: PLoS One. 2024 Mar 7;19(3):e0298601. doi: 10.1371/journal.pone.0298601 (PMC10919601; doi:10.1371/journal.pone.0298601)
Supplement: S1 Data — (ZIP) [file pone.0298601.s001.zip › Data/地级市Z_F工作报告.zip!/║╙─╧/║╫▒┌╩╨/└┤╫╘╣1⁄2╓┌║┼í╛┬φ┐╦╩2╛▌═°í┐_2017.docx]

各位代表：

　　现在，我代表市人民政府，向大会报告工作，请予审议，并请各位政协委员和其他列席人士提出意见。

　　一、2016年工作回顾

　　过去的一年很不平凡。面对严峻复杂的外部环境和艰巨繁重的工作任务，在省委、省政府和市委的坚强领导下，市政府与全市人民一道，深入贯彻习近平总书记系列重要讲话和调研指导河南工作时的重要讲话精神，主动适应引领经济发展新常态，以提升求进为总基调，以稳增长保态势、大气污染防治和扶贫脱贫三个攻坚战为重点，统筹推进改革发展稳定工作，经济社会保持平稳健康发展，较好完成了市十届人大四次会议确定的目标任务，实现了“十三五”良好开局。全市生产总值769.4亿元、增长7.9%，规模以上工业增加值449.3亿元、增长8.8%，第三产业增加值204.1亿元、增长7.4%，固定资产投资809.1亿元、增长16.8%，社会消费品零售总额205.9亿元、增长12.1%，居民消费价格指数上涨2.1%，一般公共预算收入55.6亿元、增长6%，城镇居民人均可支配收入26184元、增长6.7%，农村居民人均可支配收入14022元、增长7.9%。主要抓了以下几项工作：

　　（一）坚持稳增长保态势，经济运行总体平稳。面对经济发展新常态和较大的下行压力，坚持把稳增长保态势作为全局工作的突出任务，综合施策、精准发力、狠抓落实，固定资产投资、规模以上工业增加值等主要经济指标高于全省平均水平、位居资源型城市前列。把扩大有效投资作为关键举措。一体化强力推进开放招商、项目建设、企业服务工作，充分发挥市级领导干部为组长的12个重点产业推进组、4个驻地招商组和1个督查督导组作用，分组筛选100个项目重点推进、100条线索重点招商、100家企业重点帮扶。新落地、新签约超亿元项目109个和130个，被评为省对外开放工作先进市；重点项目完成投资占年度计划的101%，总投资165亿元的28个重点项目竣工投产，总投资629亿元的60个重点项目开工建设；企业反映的一批难点问题得到有效解决，有力保障了正常生产经营。把强化监测调度作为有效手段。围绕提高监测预警调度的针对性、灵活性和前瞻性，坚持“旬例会、月研判、季分析”制度，充分发挥农业、工业、服务业3个运行监测组作用，建立了60家重点企业直报系统和88家重点用电大户台账。围绕增强要素保障能力，组织开展银企对接、产销对接、用工对接、产学研对接，较好保障了项目和企业资金、劳动力、土地等需求，余额存贷比保持全省第一，新增各类挂牌企业14家。把完善政策措施作为重要保障。出台了促进工业稳增长调结构增效益工作方案、促进住房消费推动房地产业持续健康发展实施意见、推进金融普惠支持经济社会发展意见等政策措施，特别是针对“僵尸企业”和问题楼盘逐个制定了处置方案和盘活计划并抓好督促检查。

　　（二）坚持调结构促转型，质量效益持续提升。按照省委、省政府定位我市加快转型发展的要求，以供给侧结构性改革为主线，加快产业优化升级和城乡协同发展，有效提升了发展层次和水平，成功跻身中原城市群核心发展区。产业转型升级步伐加快。围绕做强工业，突出清洁能源与新材料、绿色食品、汽车零部件与电子电器、镁精深加工、现代家居5个重点产业，加快培育“百千亿”级产业集群，产业规模、质量和效益实现新提升，5个重点产业完成增加值432.9亿元、占规模以上工业增加值比重达到96.3%，其中高新技术产业增加值占规模以上工业增加值比重提高到31.8%。围绕做优农业，加快完善现代农业生产体系、产业体系、经营体系，持续强化信息技术支撑和综合服务保障能力，粮食总产保持稳定，种养业结构稳中趋优，农业现代化综合指数保持全省第一，农业标准化、信息化和粮食高产创建水平继续保持全国领先，成功创建全国农业社会化服务综合标准化示范市和4个生态原产地产品。围绕做大服务业，加快壮大新型物流、休闲旅游、网络经济、文化创意、健康养老等特色服务业，10多个“互联网+”新业态新模式发展成效明显，“互联网+农业”走在全国前列，“鹤壁等旅游城市和旅游集散中心建设”列入国家《“十三五”旅游业发展规划》，“石林会议”旧址入选全国红色旅游经典景区名录，五岩山成功创建国家4A级景区，浚县古城、淇河生态区、朝歌文化园、文化旅游风情街、国际购物中心等服务业项目建成开放，服务业增加值占生产总值比重稳步提高，三次产业结构为8.1∶65.4∶26.5。城乡建设进程全面提速。新型城镇化稳步推进，“一核双星多支点”单元组合型城镇化格局日益完善，综合承载能力和吸纳人口能力持续提高，农业转移人口市民化态势良好，全年转户落户城镇人口超过4万人，预计城镇化率达到56.96%，成功入选第三批国家新型城镇化综合试点。“三城联创”深入推进，国家卫生城市通过省级评审和国家暗访，国家园林城市通过省级专家初审并上报国家，省级文明城市获得提名，新增国家和省传统村落3个、省美丽乡村建设试点11个，农村人居环境达标村占比提高到65%，城乡功能面貌持续改善。城乡一体化发展政策措施、体制机制更加健全，城乡一体化示范区、示范镇等引领带动作用进一步增强，农村基础设施和公共服务设施更加完善，城乡一体发展更趋协调。

　　（三）坚持推改革抓创新，动力活力不断增强。围绕破解经济社会发展深层次矛盾和问题，加快推进基础性关键性改革，深入推进创新创业工作，改革推动和双创驱动能力持续增强。各项改革全面深化。148项重点改革事项完成或基本完成116项，一批重要领域和关键环节改革事项取得实质性进展，全国商务综合行政执法体制改革试点市荣获中期评估优秀等次，行政审批标准化、地方立法等走在了全省前列。创新创业成效明显。新增省级以上创新平台9个、总数达到78个，与5家省级以上学会开展了深度合作、总数达到17个，获批省级创业孵化示范基地3家、众创空间3家、科技特派员工作站5家。新认定高新技术企业9家，规模以上高新技术领域企业达到220家、占规模以上工业企业的37.7%。超级悬浮床工业示范装置、世界最大单口径射电望远镜反射面板、高性能无源光电子材料与器件等关键核心技术跻身世界领先水平，仕佳光子公司荣获省科技进步一等奖并被省政府推荐申报国家科技进步奖，国立光电公司连续两年荣获国家创新创业大赛一等奖。科技进步贡献率达到55%，市场主体增长超过20%，成功创建全国创新驱动示范市、全国返乡农民工创业创新示范基地，获批创建全国金属镁产品质量提升示范区。

　　（四）坚持打基础扩优势，发展势能加快积聚。抓住既利当前又利长远的重要领域，持续加力使劲，不断夯实基础、厚植优势。试点示范再创佳绩。国家级试点示范工作推进顺利，国家海绵城市建设试点和节能减排财政政策综合示范市项目完成投资37亿元、顺利通过国家年度考核验收，新增7个国家级和10多个省级试点示范名片，国家级试点市、示范市、示范区累计超过40个，试点经济继续走在全省前列，有力促进了经济社会发展。交通出行更加便捷。郑济铁路过境设站，新老区市域铁路、京港澳高速东西复线列入省规划，鹤浚快速通道、东大线改建等工程完工通车，新老区快速通道拓宽改建工程开工建设，建设农村公路266公里，累计投用新能源公交车300台、公共自行车2000辆，公交线路得到优化、班次有所增加，市民出行更加方便快捷。合作领域不断拓展。与美国杜瓦迪市、韩国首尔市瑞草区缔结为友好城市，达成各类国际合作协议与意向34个；与省交通投资集团、中原资产管理公司等投融资平台，与天津大学、省农科院等科研院校，与郑州航空港经济综合实验区、中国铁路通信信号公司等相关单位开展了战略合作。载体平台持续完善。在省重点项目暨产业集聚区建设观摩点评活动中，金山产业集聚区、商务中心区、城乡一体化示范区均获豫北五市第2名，浚县产业集聚区、商务中心区位居全省“十先”“十强”行列，产业集聚区和商务中心区实现了一星级全覆盖。环境质量有效改善。一批群众反映强烈的污染问题得到有效整治，被九三学社中央确定为新农村建设农业面源污染治理示范点。空气质量三项指标在省大气污染传输通道城市中位居前列，优良天数多出全省平均水平8天。淇河水质继续排名全省第一、汤河水质在全省排名较2015年年底前进21个位次，集中式饮用水源地水质达标率保持100%，亚行贷款1.5亿美元淇河流域环境治理与生态保护项目正式签约。森林覆盖率、城市绿化覆盖率分别达到32.6%、39.6%，云梦山成功创建国家森林公园。节能减排效果良好，预计万元生产总值能耗下降7%。

　　（五）坚持防风险惠民生，社会大局保持稳定。以防风险为重点，有效防范化解金融风险、企业风险、债务风险、环境风险和非法集资问题，着力做好安全生产、信访稳定、治安防控等工作，食品药品监督执法标准化信息化、公众安全感和满意度保持全省前列，被评为省平安建设先进市，成功应对汛期3轮大范围强降水，确保了安全度汛和群众生命财产安全。以民生工程为重点，持续加大投资力度，全市民生支出占财政支出的74.8%，重点民生工程基本完成，市人民医院综合病房楼、残疾人康复服务中心、综合活动中心、示范性综合实践基地A楼和B楼、档案馆、图书馆新馆、食品综合检测服务平台等一批重点民生项目建成投用或具备使用条件，保障性住房新开工595套、基本建成5000套，改造农村危房3452户。1.5万名贫困人口实现稳定脱贫，27个贫困村脱贫摘帽。城乡居民医保低保、城镇居民和职工医保支付比例、企业退休人员基本养老金、特困人员供养、基本公共卫生服务经费、大病保险筹资等标准持续提高。城镇教育资源持续扩容，城乡养老服务机构更加健全，城市公立医院改革进入国家联系试点市。民俗文化节、樱花文化节、淇河诗歌文化系列活动、文博会、食博会、环淇河自行车赛等较好满足了市民需求，规模档次和品牌影响持续提升。

　　一年来，我们切实加强政府自身建设，严格落实中央八项规定精神，巩固扩大“三严三实”专题教育成果，深入开展“两学一做”学习教育，持续整治懒政怠政、为官不为，不断加大反腐倡廉工作力度，风清气正、干事创业的氛围更加浓厚。我们切实加强民主法治建设，深入推进依法治市，自觉接受人大法律和工作监督、政协民主监督、社会舆论监督，支持民主党派、工商联和无党派人士参政议政，328件人大代表建议和政协提案全部办结，依法行政工作走在全省前列，被确定为全国“六五”普法先进市、全国法制宣传教育先进市、全国法治城市创建先进单位。我们切实加强精神文明建设，好人好事不断涌现，新入选一批全国文明家庭、中国好人榜、感动中原十大年度人物等，被评为全国文明经营示范城市。我们切实加强兵员征集、国防教育、国防后备力量及人民防空建设，武警鹤壁支队机动大队项目基本建成，驻鹤解放军、武警部队和民兵预备役为地方发展做出重要贡献，军民融合深度发展，双拥工作持续深化。妇女儿童、残疾人、红十字、慈善等事业健康发展，民族宗教和外事侨务、对台、审计、统计、档案、史志、气象、地震、人防等工作取得了新成绩。

　　各位代表！总体上看，去年我市呈现出改革发展稳定更加协调统一、速度质量效益更加协调统一、经济社会生态更加协调统一、产业城市乡村更加协调统一的良好态势，为率先全面小康、建设品质“三城”打下了更为坚实的基础。以上成绩是在面对严峻复杂形势和重重风险挑战中取得的，是省委、省政府和市委正确领导的结果，是市人大和市政协大力支持的结果，更是全市人民拼搏奉献的结果。在此，我代表市人民政府，向全市人民，向各民主党派、各人民团体和各界人士致以崇高的敬意，向关心支持鹤壁发展的各界朋友表示衷心的感谢！

　　在总结成绩的同时，我们也清醒认识到，经济社会发展中还存在一些困难、问题和挑战：一是经济增长稳中向好的基础仍不稳固，下行压力依然较大，科技创新和转化能力不强，要素供给愈加趋紧，部分行业企业生产经营困难，需要加快动力转换，继续稳增长保态势；二是经济结构不优、发展层次不高的问题没有根本改善，尤其是服务业发展相对滞后、城乡协同能力较弱，企业效益、财税收入和居民增收动力不足，需要提高质量效益，继续调结构促转型；三是维护社会和谐稳定压力仍然较大，利益群体和诉求更趋多元复杂，生态环境治理与人民群众期盼还有不小差距，民生保障改善任务依然艰巨，需要创新社会治理，继续防风险惠民生；四是政府职能转变还不到位，社会治理水平有待提高，个别公务人员作风不实、服务不优、效能不高、为政不廉，需要打造最优环境，继续加强政府自身建设。对此，我们一定高度重视，进一步强化责任担当，积极采取有效措施加以改进解决。

　　二、2017年工作安排

　　今年是党的十九大召开之年，是实施“十三五”规划的重要一年，是推进供给侧结构性改革的深化之年，也是率先全面小康、建设品质“三城”的关键之年。综合判断，当前我市发展机遇与挑战并存、压力与动力同在，处于可以大有作为的战略机遇期，处于转型发展、升级突破的紧要关口，只要我们保持战略定力、增强发展自信，紧紧抓住用好有利条件，积极妥善应对复杂局面，就一定能够掌握发展主动权、开辟发展新境界。

　　今年政府工作的总体要求是：深入贯彻党中央治国理政新理念新思想新战略，全面落实省委、省政府和市委各项决策部署，适应把握引领经济发展新常态，坚持转型发展，以提高发展质量和效益为中心，以推进供给侧结构性改革为主线，以增进人民福祉为出发点和落脚点，着力发挥优势打好“四张牌”，着力统筹打好“三个攻坚战”，着力组织打好“六项行动组合拳”，加快构建现代产业、现代农业、现代城乡、现代生态、现代治理新体系，谱写率先全面小康、建设品质“三城”新篇章，以优异成绩迎接党的十九大胜利召开。

　　今年经济社会发展的主要预期目标是：生产总值增长8%、在实际工作中争取更好结果，规模以上工业增加值增长8.5%，全社会固定资产投资增长14%左右，一般公共预算收入增长7.5%，居民人均可支配收入与生产总值增长同步，社会消费品零售总额增长12%，城镇新增就业2.7万人以上，居民消费价格涨幅3%左右，常住人口城镇化率完成国家新型城镇化综合试点任务，节能减排完成国家节能减排财政政策综合示范市要求，环境质量改善完成省下达目标。

　　落实总体要求，实现预期目标，需要全面贯彻落实市委九届二次全会和市委经济工作会议精神，以产业转型、结构转型、城乡转型为方向，以转型发展、扶贫脱贫、环境保护攻坚战为重点，以开放招商、项目建设、企业服务为抓手，以改革推动、双创驱动、试点带动为动力，以民主法治、社会治理、最优环境为保障。突出“一个主题”，就是转型创新、提升求进，切实把这一主题作为今年经济社会发展的努力方向和动力所在，以工作标准和工作实效的全面提升带动全局工作的高质量进步；把握“五个注重”，就是注重保持定力、抓住关键、遵循规律、落实责任、党建引领，全力把既定思路、目标、举措抓实抓好、抓出成效；处理“六个关系”，就是处理好点与面、好与快、新与旧、大与小、远与近、内与外的关系，确保经济社会持续平稳健康发展。

　　（一）着力扩大有效投资，持续稳增长保态势。继续一体化推进开放招商、项目建设、企业服务，着力重点产业、关键领域和民生工程，持续激发和释放投资潜力。深化开放招商。突出“引进来”，持续强化链条式和集群式专题招商，围绕优势特色产业，选准重点区域，广泛借智借力，重点推进100个重点招商项目，积极跟进汉能移动能源产业园、采煤沉陷区光伏发电领跑技术基地、年产180万吨甲醇制烯烃等项目，着力引进一批先进科技成果转化和产业试验中试项目，支持各类企业及科研院校等在我市设立研发中心、生产基地、结算中心和地区总部等。大力“走出去”，积极融入国家“一带一路”建设和省相关国家战略规划，抓住进入中原城市群核心发展区机遇，突出抓好与郑州航空港经济综合实验区的战略合作、与国内各口岸的关检合作、与国际友好城市的全方位合作，完善提升现有开放平台功能，积极申建鹤壁综合保税区和各类出口基地，加快培育综合贸易服务平台，不断壮大外向型经济。根据国务院允许地方政府在法定权限范围内制定出台招商引资优惠政策的导向，及时出台更具针对性、实效性和可操作性的政策措施，确保实际利用省外资金增长7%、实际利用境外资金超过8.1亿美元、进出口总额超过2.3亿美元。抓好项目建设。以提高项目开工率、资金到位率、投产达效率和带动形成的投资量、利税量、就业量为重点，着力抓好120个重点项目建设，力争全年新开工项目60个、竣工项目26个、完成投资320亿元以上；每个县区谋划5个超10亿元项目，力争全市新谋划储备项目150个以上，保持达产一批、建设一批、开工一批、储备一批的梯次发展格局。改进企业服务。完善企业服务长效机制，选择一批重点骨干企业、较大规模的民营企业、有特色有潜力的小微企业和初创企业，提供分类、精准、系统、到位的服务。畅通政企沟通渠道，构建“亲”“清”政商关系，加快推动服务窗口由接收向办事窗口转变。稳妥处置“僵尸企业”，有效激活问题楼盘、停产半停产企业和闲置土地厂房等资源。弘扬企业家精神和工匠精神，加大培训和引导支持力度，以企业家素质提升引领产业素质提升。优化要素供给。以优化信贷投放结构和提高直接融资成效、规模为重点，着力破解实体经济资金短缺难题。以探索工业弹性供地机制和提升存量土地利用效率为手段，着力提高产出效益。以引进培养高层次管理人才、技能人才、科研人才等为方向，着力缓解结构性用工矛盾。

　　（二）着力提高供给质量，持续推进产业转型。加大供给侧结构性改革力度，打好以稳增长保态势为主的转型发展攻坚战，持续做强工业、做大服务业，不断增强产业整体素质和核心竞争力。持续抓好“去降补”。以煤炭行业去产能为重点，再退出煤炭产能60万吨，坚决防止已化解的过剩产能死灰复燃，坚决用市场、法治的办法和环保约束淘汰落后产能。以房地产去库存为重点，有效缩短商品住房去化周期，努力提高供应品质、扩大住房消费。以优化融资结构和拓宽融资渠道为重点，扎实推进国有投融资机构去杠杆工作，切实提升金融服务实体经济的质量和效率，持续优化企业资产结构。严格落实降成本政策措施，加大减税、降费、降要素成本工作力度，深入清理规范基金和收费，推动企业降本增效。着力补齐产业结构优化、城乡一体化发展、基础设施完善、公共服务提升、扶贫脱贫攻坚等方面的短板，优化软硬件环境。加快工业转型升级。实施“百千亿”产业集群提升工程，加快建设150万吨煤焦油综合利用等总投资327亿元的50个重点项目、确保完成投资127亿元以上，力争清洁能源与新材料产业主营业务收入达到1100亿元、绿色食品产业达到650亿元、汽车零部件与电子电器产业达到270亿元、镁精深加工产业达到60亿元、现代家居产业达到200亿元。实施制造业创新能力提升工程，抓好100个先进制造业改造提升项目，推进一批带动作用强的工业示范项目。制定实施中国制造2025鹤壁行动纲要，推进“互联网+协同制造”，培育建设一批智能工厂、智能车间。实施增品种、提品质、创品牌专项行动，引导支持企业加强质量和品牌建设，推进产品向中高端迈进。加快产业集聚区提质转型发展，努力实现争先晋级，积极创建省级及以上经济开发区、国土集约节约示范园区等。提速发展现代服务业。坚持改造提升传统服务业与培育引进新兴服务业并重，力争第三产业增加值增长9%，加快缩小服务业占比与省平均水平差距。充分发挥南太行旅游集散地作用，推进休闲旅游、文化创意与健康养老互动融合发展，大力发展新型物流业，加快壮大网络经济，重点抓好煤炭物流储配加工基地、旅游综合体、跨境电商产业园等总投资263亿元的31个重点项目建设，确保完成投资102亿元以上。进一步放宽服务业准入限制，大力发展金融服务、云计算、大数据等新业态新模式，打造一批特色服务业集群。全面提升商务中心区发展水平、打造高成长性服务业发展高地，持续拓展特色商业区生活性服务功能、打造生活性服务业集中区，积极做好各类服务业专业园区规划建设和省级专业园区、示范园区申报工作，确保服务业“两区”项目完成投资50亿元以上。

　　（三）着力破解“三农”难题，持续促进增效增收。以绿色生态可持续为导向，深化农业供给侧结构性改革和农村改革，有效促进农业增效、农民增收、农村增绿，保持现代农业发展水平在全省、全国的领先地位。大力发展现代农业。高标准建设国家整市建制现代农业示范区和现代农业园区，积极做好全国粮食绿色高产高效示范市、全国畜牧业绿色发展示范市、生态原产地产品保护示范市、国家食品安全城市创建工作。深入推动一二三产融合发展，抓好鹤浚快速通道都市生态农业产业带、建业绿色基地农业综合体、中鹤现代农业观光等重点项目，做好浚县全国农村产业融合发展试点示范县建设。持续提升农业农村信息化水平，完善中国智慧农业研究院、农业硅谷产业园、农业大数据中心和“互联网＋农业平台”服务功能。着力调优种养结构，扩大优质小麦、优质花生、优质畜禽、优质林果等规模，推进农业生产绿色化、标准化、品牌化，扩大无公害、绿色、有机和地理标志优质农产品供给。积极推进农村改革。全面完成农村集体土地的所有权、宅基地使用权和集体建设用地使用权的确权登记发证工作，落实承包土地所有权、承包权、经营权分置政策。健全县乡农村经营管理体系，深入开展承包土地经营权抵押贷款，稳妥推进农村集体经营性建设用地入市和土地征收制度改革，引导土地经营权依法规范有序流转，有效激发农村活力、调动农民积极性。加快浚县国家农村承包土地经营权抵押贷款试点和省农村产权制度改革试点建设。健全完善金融支持三农政策体系，不断提升农村金融服务水平。着力增加农民收入。以农业产业化龙头企业、农民专业合作社、家庭农场、新型职业农民为重点，培育壮大新型农业经营主体和社会化服务主体。加快农业信息化进村入户工程建设，积极发展农村电商、乡村旅游等新业态新模式，提升农产品品质、品相、品牌，提高农产品加工转换率，增加农民经营性收入。加强农民职业培训，提高就业能力，增加农民务工收入。落实惠农补贴政策，增加农民转移性收入。扩大农业保险产品种类和覆盖范围，有效帮助农民避灾减损。深入开展脱贫攻坚。坚持精准扶贫、精准脱贫，牢牢抓住增加贫困人口收入这个中心环节，统筹优化推进转移就业、产业扶持、易地搬迁、社会保障、特殊救助五大扶贫举措，确保27个贫困村摘帽、1.1万名农村贫困人口实现稳定脱贫、1215名贫困群众易地扶贫搬迁。

　　（四）着力统筹城乡发展，持续提高协同能力。主动对接国家促进中部地区崛起“十三五”规划、中原城市群规划等，以国家新型城镇化综合试点、国土空间规划优化和省百城建设提质工程实施为重点，加快推进新型城镇化和城乡一体化。优化城乡空间布局。统筹规划建设淇滨区、开发区、城乡一体化示范区，加快淇滨区都市功能区建设和鹤淇一体化发展，实施好老城区统筹提升发展年度计划和淇县百城建设提质工程，加快山城区资源型城市转型发展先行区和鹤山区综合改革实验区建设，有序实施浚县古城保护建设提升和新城发展，加快实现城区功能统筹提升。推进百强乡镇、特色小镇、宜居小镇、美丽乡村、传统村落等建设申报工作，提升镇域经济、乡村经济，持续改善农村生产生活条件和乡村环境面貌。

　　促进农业人口转移。完善城乡一体化示范体系，高标准建设城乡一体化示范区和示范镇，着力增强示范带动能力。落实完善农业转移人口市民化“1+10”政策体系，强化“一基本两牵动三保障”，深化户籍制度改革，推动居住证制度落地，加大农民转移就业培训力度，不断提升农民工转化为市民的能力，持续提高常住和户籍人口城镇化率。提升综合承载能力。坚持中心城区带动，完善城市基础设施，确保21个重点基础设施项目完成投资57亿元以上，持续改善市民生产生活条件。积极做好郑济铁路协调服务和市域铁路、107国道改线等前期工作，加快推进新老区快速通道拓宽改建、鹤辉高速建设、南海路改建等，建设农村公路230公里，统筹抓好市政道路、交通信号灯与标识、城市停车场、公交场站、充电桩等建设，优化增设公交路线和站点。谋划推进城市地下综合管廊和一批水电路气暖、垃圾污水处理、消防等基础设施建设，实施农村电网改造、沼气建设、饮水安全等工程，做好南水北调工程向老区供水前期工作。持续扩大新老城区、两县县城集中供暖面积，力争重点区域、关键区域实现全覆盖。加快推进淇县县城污水处理中心、浚县第二污水处理厂等扩容增量，提高中水利用水平，积极推进老城区雨污分流改造工程。改进城市规划管理。启动城市修补和生态修复工作，谋划好新一轮城市总体规划修编工作。以建设国家和省级文明城市为契机，加大联合执法力度，持续提高人居环境质量。推进城市执法体制改革，优化整合市县区政府城市管理机构，新老城区全面实行网格化管理，加快新型智慧鹤壁及浚县、淇县数字化城市管理系统建设，不断提高城市精细化管理和信息化服务水平。

　　（五）着力深化改革创新，持续加快动能转换。围绕破解经济社会发展深层次矛盾和问题、更好发挥改革推动和创新驱动作用，积极推进基础性关键性改革和双创驱动发展，持续加快发展动能转换。持续全面深化改革。着力扩大改革受益面，进一步激发市场活力和社会创造力。深化行政改革。重点抓好“放管服”改革，全面推行权力、责任和效率清单，继续精简行政审批事项、清理规范中介服务，提升行政审批标准化试点水平。加快商事制度改革，巩固“五证合一”“两证整合”改革成果，推动“双随机、一公开”监管全覆盖，继续开展减证便民专项行动。加快推进“互联网+政务服务”，着力提高网上申报办件比率，年底前基本建成网上政务服务平台，实现网上行政审批省、市、县、乡四级全覆盖，重点便民服务事项实行“一号申请、一窗受理、一网通办”。深化投融资体制改革。加快政府投融资平台市场化转型步伐，更好发挥政府产业基金、投资基金的撬动和杠杆作用，持续扩大政府与社会资本合作模式的规模和范围，着力提高融资能力、效率和效益。探索建立民营企业项目融资推介长效机制，有效扩大社会投资特别是民间投资，确保民间投资增速不低于固定资产投资增速。深化财税体制改革。健全财力稳定增长机制，既注重财源涵养又坚持依法治税，着力提高税收占财政收入的比例，持续优化财政收入结构。深化预算管理制度和财政资金投入机制改革，创新财政支持方式方法，用足用活上级政策，调整优化支出结构，通过整合、盘活、统筹预算内外等多种方式和渠道，增强市县区基本保障水平。抓好税收征管体制改革任务落实，建立完善地税征收、财政管理、社保发放、审计监督的社会保险费征管新机制。深化国有企业改革。完成“三供一业”维修改造保供、社区管理组织移交、省定国有企业退休人员社会化管理任务。深化市属国有企业改革，有序推进混合所有制改革，着力改革企业产权制度、完善法人治理结构、创新国有企业负责人选拔制度和薪酬制度、健全国有资产监管方式，有效增强国有经济和国有企业动力活力。深化市场配置要素改革。大力发展非公有制经济，继续清理限制非公有制经济发展的不合理规定，更好保护各种所有制经济产权和合法权益，鼓励支持引导非公有制经济在公共服务产品供给领域发展。围绕金融业、物流业、电子商务、建筑业、政府与社会资本合作等重点领域，鼓励引导市场经营主体在我市设立独立法人机构。鼓励有条件的集聚区发起组建售电公司，打捆参与电力直接交易，有效降低用电成本。统筹抓好其他领域改革。持续推进创新驱动。以创建国家创新型城市为目标，以科技创新为重点，统筹推进产品创新、管理创新、模式创新、组织创新，加快培育发展新动能。完善创新政策。以深化科技体制改革、激发创新活力为导向，出台建设创新型城市实施意见，制定企业科技创新补助、投入产出考核评价等配套政策措施，健全科技型企业技术创新贷款风险补偿机制，做好科技保险和知识产权质押贷款试点推广工作，加快建立以财政投入为引导、企业投入为主体、金融市场为支撑的多元化科技投入体系，确保全社会研发投入占生产总值比重、财政科技投入占公共财政支出比重不低于全省平均水平。搭建创新平台。全面提升国家可持续发展实验区、国家农业科技园区、鹤壁高新技术产业开发区发展水平，加快建设国家汽车电子和光电子检测检验中心、市科技创新创业服务中心、创新驱动成果转化分中心、高科技创业园孵化园等服务平台，在现有创新平台功能作用发挥基础上，力争新建3个以上国家级创新平台。壮大创新主体。实施好产业技术提升、骨干龙头企业强壮、科技型中小企业培育等工程，新建一批产业技术创新战略联盟、产业技术研究院、院士工作站、众创空间等，着力增强企业创新、产业创新、协同创新等能力。充实创新人才。突出“高精尖缺”导向，完善更加开放有效的人才引进政策，引导企业加强与科研院所和高等院校的合作，扎实做好“兴鹤聚才计划”“人才回归工程”等，着力培育引进科技创新领军人才、创新团队。

　　（六）着力优化生态环境，持续建设美丽家园。牢树并落实绿色发展理念，加大环境保护投入力度，加快构建完善的环境治理体系，切实增强环境治理能力，努力实现更高质量、更可持续的发展。坚决打赢环境保护攻坚战。全面深化大气污染防治攻坚，提升科学治污、依法治污、全民治污、联防治污水平，确保优良天数211天以上、可吸入颗粒物（PM10）年均浓度每立方米108微克以下、细颗粒物（PM2.5）年均浓度每立方米63微克以下。全面打响水污染治理攻坚，推行“河长制”，坚持一河一策、水陆统筹，确保淇河水质保持全省第一，卫河、汤河、思德河水环境质量达标，基本消除建成区黑臭水体，河流断面水质达标，集中式饮用水水源安全；实行最严格水资源管理制度，加快建设节水型城市，高标准完成全国海绵城市建设试点市任务，力争省级水生态文明建设试点市通过验收。加强土壤污染防治，实施好新农村建设农业面源污染治理示范项目，启动土壤污染状况详查，扎实推进土壤污染治理修复、农业农村污染防治和农村环境综合整治工作。加强环境建设和生态修复。统筹推进森林、湿地、流域等生态系统修复建设，组织开展以“三城联创”“三廊三带三区”建设为重点的生态文明建设提升专项行动计划，统筹实施好亚行贷款淇河流域环境治理与生态保护工程，全面完成新城区水系生态治理工程，扎实推进国家湿地公园、示范区湿地公园、盘石头水库坝后区域综合保护利用、老城区采煤沉陷区修复改造，开工建设淇河植物园、淇河南森林生态带、淇河风貌带东延等项目，完成造林和补植补造6.25万亩，力争国家园林城市创建成功。提升低碳循环发展水平。突出抓好节能减排项目改造、蓝天碧水工程等5大典型示范项目，完成投资15亿元，支持高耗能高排放企业实施技术改造，力争再投用新能源公交车60辆和公共自行车2500辆，确保完成国家节能减排财政政策综合示范市建设任务。落实《鹤壁市循环经济生态城市建设条例》，制定建筑废弃物、餐厨废弃物和生活垃圾的收集、运输和处置管理办法，做好循环经济示范企业和示范园区建设，发展节能环保和再生资源综合利用产业，推进静脉产业园和生活垃圾焚烧发电、建筑垃圾资源化利用、农作物秸秆综合利用等项目，健全循环经济标准体系和产业链条、保持全国领先地位，力争宝山循环经济产业集聚区建成省级循环经济示范园区。

　　（七）着力发展社会事业，持续改进公共服务。加快完善基本公共服务体系，努力满足人民群众多样化需求，抓好总投资24亿元的9个重点社会事业项目，完成投资12亿元以上，确保示范性综合实践基地C楼（城展馆、科技馆、美术馆）和全民健身活动中心暨体育公园主体完工。努力办好人民满意的教育。以提高质量和促进公平为重点，以实现基本公共教育服务均等化、基本实现教育现代化为目标，持续深化教育体制改革。大力发展学前教育，启动第三期学前教育三年行动计划，新建、改扩建幼儿园10所，公办幼儿园和普惠性民办幼儿园覆盖率达到87%以上。均衡发展义务教育，继续实施“全面改薄”和扩充城镇义务教育资源五年计划，完成30所学校设施设备配备，改造19所学校校舍，力争天赉小学、齐贤中学、松江小学、明志中小学主体完工，全市中小学大班、超大班减少284个；抓好农村中小学校优化布局和农村寄宿制学校建设，积极整合生源少、效益低的学校开展集中办学，提高农村学校办学规模和质量。加快普及高中阶段教育，引进扩大优质教育资源，持续推进高中教育高质量发展。整合发展职业教育，突出服务地方产业发展，继续推进品牌示范院校和优势特色学科建设。落实普通高中家庭经济困难学生国家资助政策，完善“两免一补”和学校生均公用经费基准定额政策，解决好因贫失学和因学致贫问题，决不让贫困阻挡孩子求学上进的脚步。支持规范民办学校发展，提升发展特殊教育，更加注重家庭教育。全面提升健康服务水平。努力构建覆盖城乡的医疗服务体系，稳妥推进城市公立医院改革，加强乡村、社区卫生基础设施和医疗队伍建设，推广医疗服务信息化和家庭医生签约服务，逐步形成社区首诊、双向转诊、分级诊疗就医新秩序。健全基本医保稳定可持续筹资和报销比例调整机制，推行按病种付费为主、多种付费方式结合的医保支付方式改革。推进市级医疗机构与知名医院、医学院校开展合作，强化高端人才引进培养，持续推进市人民医院创建三级甲等医院，不断提高医疗服务水平。加快发展健康养老产业，积极引进健康管理、医疗保健、康复养生等项目，持续完善居家养老服务信息平台功能，确保豫北仁和医养结合养老中心主体完工。推进基本公共卫生服务均等化，加强重大疾病防控和儿童、妇女、老年人、残疾人等重点人群健康服务。完善城市基础设施和环境卫生设施，抓好重点部位管控和国家暗访反馈问题集中整治，力争国家卫生城市创建成功并形成长效管理机制。统筹做好健康教育服务、计生服务管理、体育设施完善、健身活动开展等工作。加快推进文化发展繁荣。加强精神文明建设，深化思想道德和社会诚信建设，弘扬社会主义核心价值观，继续开展群众性精神文明创建活动，叫响鹤壁“好人好事”品牌，积极争创国家公共文化服务体系示范区和省级文明城市。加快文化事业发展，扎实推进图书馆、文化馆、基层综合性文化服务中心等公共文化服务设施建设，继续实施常态化文艺演出、公共文化进基层、文化设施免费开放等惠民工程，做好文化遗产保护利用工作。创新发展文化产业，落实文化产业政策，扶持2—3家具有带动作用的龙头企业，建设一批重大文化产业项目。依托民俗文化节、文博会、食博会、樱花文化节、环淇河自行车赛等节会和赛事，构建具有鹤壁特色的区域文化体系，助推文化产业加快提档升级。

　　（八）着力改进社会治理，持续保障改善民生。坚持民生为本、民生优先，继续实施一批重点民生工程，加快构建全民共建共享的社会治理格局，努力维护社会秩序、促进社会和谐、保障人民安居乐业。稳定扩大就业创业。完善公共就业创业服务体系，实施更加积极的就业政策，突出做好高校毕业生、去产能企业分流职工、就业困难人员等重点群体就业工作，做好军转干部、退役士兵和随军家属的接收安置工作，继续提高农村劳动力就近就地就业比例，动态消除“零就业家庭”。大力支持大学生和返乡农民工等群体创业发展，提高创业带动就业能力。健全完善社会保障。实施全民参保登记计划，全面完成机关事业单位养老保险制度改革，调整企业和机关事业单位退休人员基本养老金，建立基本养老金调整机制，城乡居民基本养老保险基础养老金每人每月最低标准提高到80元，落实企业年金和职业年金基金管理办法。全面实施统一的城乡居民医保制度，财政补助标准提高到每人每年450元。完善城乡居民大病保险制度，落实困难群众大病补充保险制度。城乡低保财政月人均补差水平分别提高到不低于250元和142元，社会散居和机构供养孤儿养育标准分别提高到每人每月700元和1100元。落实困难残疾人生活补贴和重度残疾人护理补贴制度，完善失业保险制度，健全社会救助、社会福利和社会养老服务体系，加强农村“三留守”人员关爱服务和困境儿童保障，切实兜住民生底线。基本建成1.2万套保障性安居工程项目，完成5409套公租房分配和1000户农村危房改造，确保60%以上的新型农村社区遗留问题得到整改。加强创新社会治理。始终保持对违法犯罪活动严打高压态势，坚持打击、防范、治理并举，依法惩治黑恶势力、严重暴力、“两抢一盗”、电信诈骗等严重影响人民生命财产安全的犯罪，提高人民群众安全感。加快推进社会治安防控体系建设，以现代科学技术为引领，以基层基础为保障，统筹协调各方面力量，扎实推进人防物防技防建设，充分发挥村、社区组织作用，广泛开展群防群治，真正形成疏而不漏的“天罗地网”。加强重点行业和重点部位的安全管理，强化学校、养老院、医院、商场、车站等人员密集场所的安全防范，坚决防止发生重特大案事件和群死群伤事故。加强道路交通和消防安全，突出源头治理，狠抓现场执法，严防发生重特大火灾、交通事故。持续加强安全生产，深入推进危险化学品、非煤矿山、建筑施工等重点行业安全专项治理，加强食品药品安全监管，突出抓好重点地区、重点企业、重点环节和重要岗位，增强事故防控能力，坚决遏制较大和重特大安全事故发生。强化信访稳定工作，深入排查化解社会矛盾，有效防范化解政府和企业债务风险，建立金融风险防控预警平台，加强资产管理、企业互保等领域风险防控，扎实做好非法集资隐患排查整治、日常管控和宣传教育，依法加快已发案件化解处置进度，着力控制增量、减少存量。加强防灾减灾救灾，推进突发事件预测预警预防和应急处置体系建设。

　　加强国防教育和国防后备力量建设，支持国防和军队改革，支持驻鹤解放军、武警部队、民兵预备役和人民防空建设，深入开展双拥共建，推进军民融合深度发展，巩固和发展军政军民团结。促进民族团结、宗教和睦。支持工会、共青团、妇联、工商联等人民团体发挥桥梁纽带作用。继续做好外事侨务、对台、审计、统计、档案、史志、气象、地震、人防等工作。

　　三、加强政府自身建设

　　人民满意是政府工作最高标准。必须持续提升政府治理能力和水平，切实增强政府的执行力和公信力，努力建设人民满意的创新政府、法治政府、廉洁政府和服务型政府。

　　（一）严守纪律规矩，确保令行禁止。严格遵守《关于新形势下党内政治生活的若干准则》《中国共产党党内监督条例》等，始终保持政治定力，着力增强政治意识、大局意识、核心意识、看齐意识，在思想上政治上行动上同以习近平同志为核心的党中央保持高度一致，做到党中央提倡的坚决响应、党中央决定的坚决执行、党中央禁止的坚决不做。在省委、省政府和市委的领导下，坚决拥护和认真贯彻执行党的路线方针政策，把党中央的各项决策部署落到实处，以实际行动确保政令畅通。

　　（二）牢树“新理念”，加快转型发展。树立与时代进步和实践发展相适应的思维方式，自觉做到崇尚创新、注重协调、倡导绿色、厚植开放、推进共享。加强学习型政府建设，持续强化政府系统工作人员的知识更新和实践锻炼，坚决摒弃和破除不适应不符合新发展理念的思想认识和行为做法，不断提高理论素养、专业技能和工作水平。更加注重学以致用，将新发展理念融入到政策制定、工作安排、任务落实的各个领域和环节，助推转型创新、提升求进发展。

　　（三）弘扬法治精神，推进依法治市。全面贯彻法治政府建设实施纲要，完善法治政府建设推进机制，把政府活动全面纳入法治轨道。加大普法宣传教育，增强尊法学法守法用法意识，提高法治素质和法治能力，自觉运用法治思维和法治方式推进改革发展稳定。严格按照法定职责和程序办事，深入推进依法科学民主决策，提升服务型行政执法水平。自觉接受人大法律和工作监督、政协民主监督和社会舆论监督，拓宽参政议政渠道，切实办理好人大代表建议和政协委员提案。深化政务公开，让权力始终在法治轨道和阳光下运行。

　　（四）践行为民宗旨，做到勤政廉洁。坚持把人民放在心中最高位置，时刻牢记权力来自人民，只能用来为人民办实事、解难题、添福祉，自觉做到依法用权、公正用权、廉洁用权，让人民赋予的权力始终用来为人民服务。全面落实从严治党主体责任和“一岗双责”，严格执行中央八项规定精神和廉洁自律准则，持续强化正风肃纪，严格问责追究，重拳整治侵害群众利益的不正之风和腐败问题。继续加强勤政廉政教育，牢固树立过紧日子思想，厉行节约，反对浪费，始终保持为民务实清廉本色。

　　（五）崇尚务实重干，勇于担当作为。持续加强作风建设，进一步压缩会议、精简文件、改进督查、减少陪同、简化接待，注重统筹协调，将更多的时间和精力投入到干事创业、为民造福中去。持续抓好工作落实，改进绩效考核办法，完善督促检查制度，着力提高执行能力和行政效能，深入查处懒政怠政和为官不为行为，努力做到守土有责、守土负责、守土尽责。持续强化正向激励，严格落实容错免责办法，营造让想干者有奔头、敢干者没顾虑、干成者得重用的氛围，着力激发广大干部职工奋发有为、干事创业热情，形成强大工作合力。

　　各位代表，实干成就梦想，奋斗赢得未来！让我们紧密团结在以习近平同志为核心的党中央周围，在省委、省政府和市委的坚强领导下，继续解放思想、开拓创新，实干争先、奋力前进，全面完成各项目标任务，为我市率先全面小康、建设品质“三城”不懈奋斗，为全省决胜全面小康、让中原更加出彩做出新的更大贡献！
